# Selfish, sharing and scavenging bacteria in the Atlantic Ocean: a biogeographical study of bacterial substrate utilisation

**DOI:** 10.1038/s41396-018-0326-3

**Published:** 2018-12-07

**Authors:** Greta Reintjes, Carol Arnosti, B. Fuchs, Rudolf Amann

**Affiliations:** 1 Department of Molecular Ecology, Max Planck Institute for Marine Microbiology, Bremen, Germany; 2 Department of Marine Sciences, University of North Carolina-Chapel Hill, Chapel Hill, NC USA

**Keywords:** Environmental microbiology, Microbial ecology, Microbial ecology, Marine microbiology, Microbial ecology

## Abstract

Identifying the roles played by individual heterotrophic bacteria in the degradation of high molecular weight (HMW) substrates is critical to understanding the constraints on carbon cycling in the ocean. At five sites in the Atlantic Ocean, we investigated the processing of organic matter by tracking changes in microbial community composition as HMW polysaccharides were enzymatically hydrolysed over time. During this investigation, we discovered that a considerable fraction of heterotrophic bacteria uses a newly-identified ‘selfish’ mode of substrate processing. We therefore additionally examined the balance of individual substrate utilisation mechanisms at different locations by linking individual microorganisms to distinct substrate utilisation mechanisms. Through FISH and uptake of fluorescently-labelled polysaccharides, ‘selfish’ organisms were identified as belonging to the *Bacteroidetes*, *Planctomycetes* and *Gammaproteobacteria*. ‘Sharing’ (extracellular enzyme producing) and ‘scavenging’ (non-enzyme producing) organisms predominantly belonged to the *Alteromonadaceae* and SAR11 clades, respectively. The extent to which individual mechanisms prevail depended on the initial population structure of the bacterial community at a given location and time, as well as the growth rate of specific bacteria. Furthermore, the same substrate was processed in different ways by different members of a pelagic microbial community, pointing to significant follow-on effects for carbon cycling.

## Introduction

Marine heterotrophic bacteria transform and remineralize a large fraction of the organic matter produced in the oceans, and thus play a significant role in the global carbon cycle [1, 2]. Much of the organic matter available to marine microorganisms is in the form of dissolved organic matter (DOM), which consists of diverse low molecular weight (LMW) compounds, and high molecular weight (HMW) components such as proteins, polysaccharides and lipids [3]. To access the HMW fraction, microorganisms use structurally-selective extracellular enzymes to create LMW substrate that can be used for metabolism and growth [4–8].

Although knowledge of the function of marine microbial enzymes has increased rapidly in the last decade [5, 9, 10], we have only begun to glimpse the diversity of the enzymatic repertoire used by individual microorganisms [11, 12]. Additionally, marine bacteria exist as interacting assemblages, microbial communities, which are structurally variable and show distinct biogeographical distribution patterns [13, 14]. These compositional variabilities between communities also affect both the nature and magnitude of their enzymatic activity [15, 16]. These observations strongly suggest that there is a relationship between microbial community structure and enzymatic function, and that there are distinct functional differences among communities [5, 17, 18].

Our knowledge of the processing of HMW DOM by individual marine bacteria or by whole microbial communities is limited because most natural organic matter—particularly the HMW fraction—defies structural characterisation at a level that provides knowledge of secondary and tertiary structure [9, 19]. In situ measurements of organic matter degradation by microbial communities thus typically rely on either well-defined LMW DOM or structurally undefined HMW DOM as substrates [15, 20, 21], and frequently focus on bulk community activity (e.g., [21, 22]). These approaches yield insights into the community potential and compositional responses but are not able to directly associate individual bacteria with the degradation of a specific compound. Direct measurements of individual microbial activities in environmental samples—for example, using microautoradiography and FISH—are quite rare [23–25].

In the current study, we initially set out to investigate potential changes in microbial community composition as specific HMW substrates, polysaccharides, were enzymatically hydrolysed over time at different locations in the Atlantic Ocean. During our investigation, we serendipitously discovered a previously unrecognised mode of substrate processing in the marine environment: ‘selfish’ uptake [26]. Selfish organisms use surface-associated enzymes to bind and partially degrade HMW substrate with little to no loss of substrate to the external environment [27]. Our examination of samples from five provinces of the Atlantic Ocean demonstrated that this mode of substrate uptake is widespread, with up to 26% of total cells carrying out ‘selfish’ uptake of specific polysaccharides [26].

**Fig. 1:**
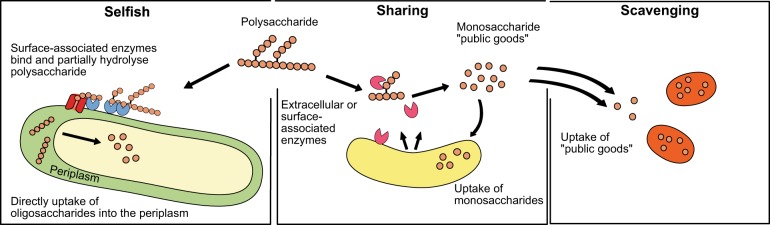
Schematic diagram of three main mechanisms of HMW substrate utilisation. Selfish: cells use surface associated enzymes to bind and partially degrade polysaccharides, which are directly taken up into the periplasm for further degradation with little to no production of extracellular hydrolysis products. Sharing: cells use surface-associated or ‘free’ extracellular enzymes to degrade polysaccharide to sizes suitable for uptake. Causes production of extracellular hydrolysis products (public goods). Scavengers: cells do not or cannot produce enzymes for the hydrolysis of polysaccharides, but take up the hydrolysis products produced by other organisms

In light of this discovery, we realised that the prevailing models of substrate utilisation among natural microbial communities must be revisited. Instead of the typical two-player models, which considers the conditions under which ‘enzyme producing-organisms’ and ‘non-enzyme producing-organisms’ (aka. cheaters) benefit from the production of hydrolysate [28, 29], we now put forward a three-player perspective, in which substrate degradation is carried out by ‘selfish’ bacteria, which transport HMW hydrolysis products into the cell, as well as by ‘sharing’ bacteria, which use cell-surface associated enzymes or freely-released enzymes to hydrolyse a substrate, yielding low molecular weight hydrolysis products in the external environment. These freely available hydrolysis products are then also available to be used by ‘scavenging’ bacteria (Fig. 1).

Adopting this new perspective, we use fluorescence in situ hybridisation (FISH), 16 S rRNA tag sequencing, and enzyme activity data to track dynamics of substrate processing within microbial communities. We compared the proportion and identities of ‘selfish’ organisms with changes in community composition during substrate hydrolysis, which enabled us to identify individual substrate utilisation mechanisms across microbial communities collected from five provinces of the Atlantic Ocean. Additionally, the activity can be linked to distinct microbial groups by analysing the change in bacterial relative and absolute abundance over time. Using these results, we investigate the manner in which phylogenetic and functional differences in microbial communities affected the turnover of marine polysaccharides.

## Materials and methods

### Sampling and substrate incubations

Seawater samples were collected from five different oceanic provinces (Northern Temperate, Northern Gyre, Equatorial, Southern Gyre and Southern Temperate; Supplementary Figure S1) as part of the Atlantic Meridional Transect (AMT) 22 cruise from Southampton, UK, to Punta Arenas, Chile. Triplicate 20 L samples were collected at each station from 20 m depth at solar noon using a Niskin bottle rosette with an attached Sea Bird CTD (Sea Bird Scientific). Six 500 ml subsamples of each triplicate were added to acid washed sterile glass bottles (18 in total) and incubated with one of six fluorescently labelled polysaccharides (FLA-PS) for a total of 12–18 days.

Single substrates (fluoresceinamine-labelled laminarin, xylan, chondroitin sulphate, arabinogalactan, fucoidan and pullulan) were added at a concentration of 1.75 µM monomer equivalent to each 500 ml incubation. These polysaccharides were selected because they are present in the oceans, and/or enzymes that hydrolyse them are widely distributed among marine bacteria [30–33]. Treatment controls consisted of a 500 ml subsample incubated in a sterile glass bottle without added polysaccharide. Six 50 ml subsamples of autoclaved seawater were incubated with polysaccharides and served as killed controls.

All bottles (18 incubations, 1 treatment control, and 6 killed controls per station) were incubated at room temperature in the dark. Subsamples for microscopy, FISH, extracellular enzymatic activity and microbial diversity were collected at regular time points (typically 5 min (referred to as T0), 1, 3, 6, 12, and 18 days). For microscopy and FISH, 20 ml of water was filtered through a 47 mm (0.2 µm pore size) polycarbonate filter, applying a gentle vacuum of <200 mbar. After drying, the filters were stored at −20 °C until further analysis. For microbial diversity analyses, 10 ml of water was filtered through a 25 mm (0.2 µm pore size) polycarbonate filter using a Whatman 420200 Swin-Lok filter holder (Sigma-Aldrich, Munich, Germany). Two ml of the filtrate from the microbial diversity sample was collected and stored at −80 °C for measurement of extracellular enzyme activity.

### Microbial diversity analysis

16 S rRNA gene sequencing was done for the biological triplicates of the chondroitin sulphate, xylan and laminarin incubations and the corresponding treatment controls. Briefly, PCR was carried out using the primers S-D-Bact-0341-b-S-17 (5′-CCTACGGGNGGCW GCAG-3′) and S-D-Bact-0785-a-A-21 (5′-GACTACHVGGGTATCTAATCC-3′) targeting the V3-V4 variable region of the 16 S rRNA [34]. The PCR amplicons were paired-end sequenced on an Illumina Miseq (Illumina, CA, USA) at the Max Planck Genome Centre (Cologne, Germany). The reads were processed using the standard settings of the bioinformatics pipeline of the SilvaNGS project [35]. The classification was done by a local BLAST search against the SILVA SSURef 119.1 NR database using blast −2.2.22+ with standard settings. A detailed description of all methodological steps and data processing is provided in the supplementary methods. All sequence data were deposited in the European Nucleotide Archive ([36]) using the data brokerage service of the German Federation for Biological Data, (GFBio [37]), in compliance with the MIxS standard [38]. The INSDC accession number for the data is: PRJEB28155.

### Extracellular enzymatic activity

Six polysaccharides (laminarin, xylan, chondroitin sulphate, arabinogalactan, pullulan, fucoidan) obtained from Sigma-Aldrich (U.S.) were labelled with fluoresceinamine (Sigma-Aldrich; isomer II), as described in Arnosti [39]. Activities of enzymes that hydrolyse each polysaccharide were determined by monitoring the changes in molecular weight of the FLA-PS over the time course of the incubations, as described in detail in Arnosti [39]. Hydrolysis of FLA-PS is detected as a change in the molecular weight distribution of the total added polysaccharide pool. Since the molecular weight resolution of gel permeation chromatography columns is somewhat limited, the earliest stages of hydrolysis—when only a small percentage of the total added pool has been hydrolysed—likely are not detected. Moreover, once most of the added polysaccharide has been hydrolysed to low molecular weight size classes, continued incubation leads to an apparent (calculated) decrease in hydrolysis rate, since there is little additional change in the molecular weight distribution of the hydrolysed polysaccharides. Note, moreover, that the added FLA-PS are in competition with naturally present substrates for the enzyme active sites; hydrolysis rates reported here are potential rates. Since the added substrate represents a ~25% increase in total DOC typical for surface ocean waters of the North Atlantic [40], however, the added substrate is likely at saturating concentrations for specific enzymes.

### Substrate staining, FISH and automated microscopy

Group specific FISH probes, including probes for the *Bacteroidetes* (CF319a), *Planctomycetes* (PLA46), *Catenovulum* (CAT653) and *Alteromonadaceae* (ALT1413, does not target *Catenovulum*) (Supplementary Table S1), were chosen based on changes in read abundances of the bacterial groups during the incubations (see Results, Substrate Related Shifts in Microbial Communities). All cell staining and microscopy was done as described in detail in Reintjes et al [26]. All cell counts and microbial abundance data are openly available at https://doi.pangaea.de/10.1594/PANGAEA.892823.

### Statistical analysis

The change in community composition over the course of each incubation was visualised using the average percentage change in abundance of each genus over time (minimum read abundance of 0.5%). This value was calculated by analysing the change in normalised read abundance of each bacterial genus over time compared to the initial community (T0), and highlighted both the positive and negative responses of each genus to the substrate addition. A detailed description is available in the Supplementary Methods.

For beta diversity analysis and related hypothesis testing, Bray-Curtis dissimilarity matrices of the total bacterial community of all samples (triplicates and treatment controls, normalised by abundance) were constructed (see Supplementary Methods for details). All statistical analyses and visualisations were performed in R using the packages Vegan, Corrplot, and Rojia [41–43]. Differences in the initial community structure between sampling sites were analysed by comparing all T0 samples (triplicates and treatment control) by analysis of similarity (ANOSIM) and visualised in non-metric multi-dimensional scaling plots (NMDS). To test for significant changes in the community composition by substrate, time, and sampling site, as well as between incubations and treatment controls, ANOSIM analyses were performed and visualised using NMDS plots. Subsequently, permutation multivariant analysis of variance (PERMANOVA) with pairwise analyses were performed to identify the amount of variance associated with individual factors (substrate, sampling site, and time).

Spearman’s rank order correlations between enzyme activities and the change in absolute abundance (FISH counts) and relative abundance (NGS sequencing) within each incubation were performed to identify the extracellularly active organisms.

## Results

### Microbial activity: extracellular hydrolysis and ‘selfish’ uptake

**Fig. 2:**
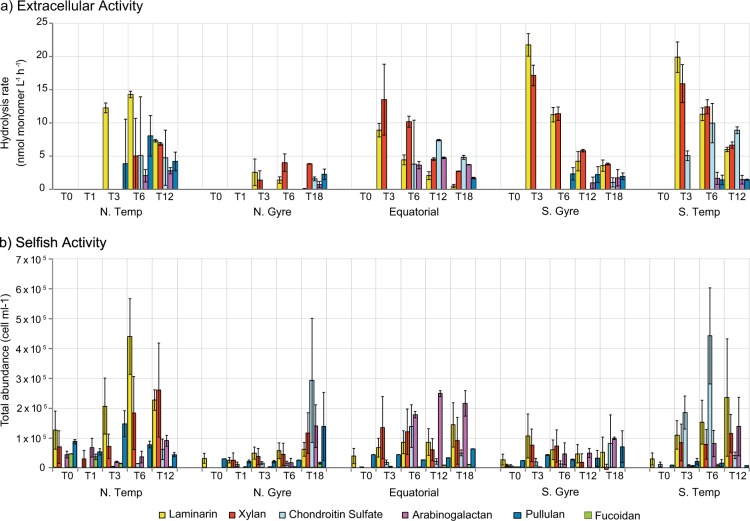
Microbial extracellular hydrolysis and selfish activity during fluorescently labelled polysaccharide incubations. **a** Hydrolysis rates of laminarin (yellow), xylan (red), chondroitin sulphate (light blue), arabinogalactan (purple) and pullulan (dark blue) in different oceanic provinces over time (0–18 days). **b** Selfish activity measured as the absolute abundance of substrate stained cells (colours; see above and fucoidan (green)) over time (0–18 days) across different oceanic provinces over time. Error bars indicate a difference in rates among biological triplicate. The selfish abundance of laminarin, xylan and chondroitin sulphate stained cells are modified from [26]

Microbial communities in surface waters of the Atlantic Ocean measurably hydrolysed five of the six added polysaccharides and showed selfish uptake of all six polysaccharides. The rates and patterns of hydrolysis, as well as the extent of uptake of FLA-PS, varied by substrate and station (Fig. 2). Laminarin and xylan were most rapidly hydrolysed at all stations, while fucoidan was not measurably hydrolysed in any incubation (Fig. 2a). The highest hydrolysis rates were measured at the S.Gyre station (22 nmol monomer L^−1^ h^−1^ and 17 nmol monomer L^−1^ h^−1^ for laminarin and xylan, respectively) and the S.Temp station (20 nmol monomer L^−1^ h^−1^ and 16 nmol monomer L^−1^ h^−1^ for laminarin and xylan, respectively; Fig. 2a). At the Equatorial, S.Gyre, and S.Temp stations, rates of laminarin and xylan hydrolysis were most rapid at day 3, and decreased with time, primarily because much of the substrate was hydrolysed to small size classes by day 3, such that longer incubation times yielded lower calculated rates (see Methods). The lowest overall hydrolysis rates were measured at the N.Gyre station: laminarin and xylan were hydrolysed (at ca. 1.6 and 3.0 nmol monomer L^−1^ h^−1^, respectively) at day 3, and other polysaccharides were measurably hydrolysed only at day 18. The N.Temp station showed a pattern of hydrolysis that differed considerably from the other stations: only laminarin was hydrolysed at day 3 (Fig. 2a), but five substrates were measurably hydrolysed at day 6, with high variability among the biological triplicates, variability only seen at this station.

The other substrates showed hydrolysis patterns considerably different from laminarin and xylan. At the N.Temp, Equatorial, and S.Temp stations, chondroitin sulphate hydrolysis was measurable from either day 3 or day 6, with the highest and earliest rate measured at the S.Temp station (10 nmol monomer L^−1^ h^−1^; T6; Fig. 2a). The rates of chondroitin hydrolysis were considerably lower at the N.Gyre and S.Gyre stations (1 nmol monomer L^−1^ h^−1^), and hydrolysis could only be measured after 18 days. Rates of arabinogalactan and pullulan hydrolysis, although measurable, were comparatively low at most stations, with the exception of the N.Temp (pullulan) and Equatorial stations (arabinogalactan).

Selfish activity, measured by the abundance of substrate stained cells, showed a somewhat different pattern than measurements of extracellular hydrolysis. Selfish uptake was measurable with every substrate—including fucoidan—at every station and frequently occurred before extracellular hydrolysis was measured (Fig. 2b). Laminarin was particularly notable in this respect, since it was taken up after just 5 min (T0) at all stations; the number of laminarin labelled cells also increased steadily with time at every station except the S.Gyre (Fig. 2b). The extent to which other substrates were taken up varied considerably by the station, but selfish uptake was widespread (Fig. 2b). At the N.Temp station, uptake was markedly rapid: 5 of 6 substrates were taken up in the first few minutes of incubation (T0). The number of substrate stained cells increased further until day 6–12 in the laminarin, xylan, and pullulan incubations, whereas the number of cells stained with fucoidan decreased. The N.Gyre and S.Gyre stations both showed lower overall selfish activity, although at the N.Gyre station, selfish uptake of xylan, arabinogalactan, pullulan, and chondroitin sulphate increased at 18 days. The Equatorial station had the highest selfish uptake of arabinogalactan, and the S.Temp station had the highest selfish uptake of chondroitin sulphate, both showing a sharp increase after 6 days (Fig. 2b).

### Substrate related shifts in microbial communities

To determine if variations in microbial community composition between stations affected the patterns of extracellular substrate hydrolysis and selfish uptake, we analysed the change in relative and absolute microbial abundances over time in the incubations. All biological triplicates of each incubation were examined to obtain a robust analysis. However, to have a manageable number of samples, we focused on a select subset of incubations. We chose to carry out further investigation on the laminarin, xylan, and chondroitin sulphate incubations, which showed the highest activity across all five stations (Fig. 2a, b). By analysing biological replicates using NGS, a statistical examination of the reproducibility of the change in community composition could be carried out, an essential factor when investigating the extent to which results are representative of the environment [44]. Moreover, incubations could be affected by grazing or by viral lysis, stochastically changing community composition independent of substrate conditions. Careful analysis of biological triplicates provides a measure of variability that might be due to such factors. We additionally counted individual bacterial groups using FISH to determine if the relative changes in abundance corresponded to changes in the absolute abundance; this analysis showed that while the absolute abundance results agreed with the patterns of change in relative read abundance they were not quantitatively comparable.

### Initial community composition and subsequent substrate dependent changes

The initial microbial community of each station was compositionally similar—dominant genera included the SAR11, SAR86, *Prochlorococcus*, SAR116, AEGEAN−169 marine group, *Alteromonas*, *Rhodobacteraceae*, NS5, NS4, and *Candidatus* Actinomarina (Supplementary Figure S2), consistent with other studies of surface ocean waters [45, 46]—but showed statistical differences (ANOSIM R = 0.48, *p* < 0.001, Supplementary Figure S3). Pairwise permutations revealed that the difference were most significant in the N.Temp and N.Gyre stations. The N.Gyre station had a higher relative read abundance of *Gammaproteobacteria* and *Alphaproteobacteria*, whereas the N.Temp station exhibited a high relative abundance of *Bacteroidetes* and *Alphaproteobacteria*, specifically *Rhodobacterales*, and a lower abundance of *Gammaproteobacteria* (Supplementary Figures S2 & S4).

**Fig. 3:**
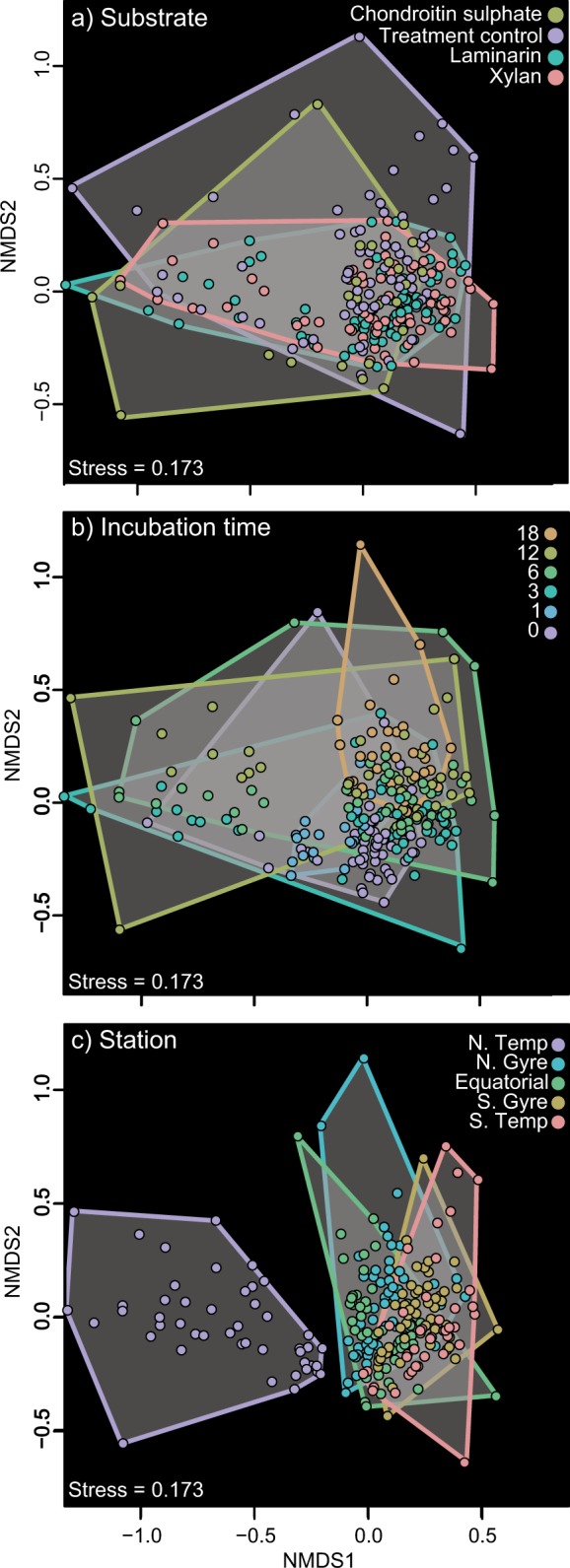
NMDS plots showing Bray-Curtis dissimilarity in community composition **a** across substrate incubations, **b** over time (0 to 18 days) and **c** across stations. The communities of a specific treatment are colour coded and connected by grey boxes with colour coded borders (see legends)

**Fig. 4:**
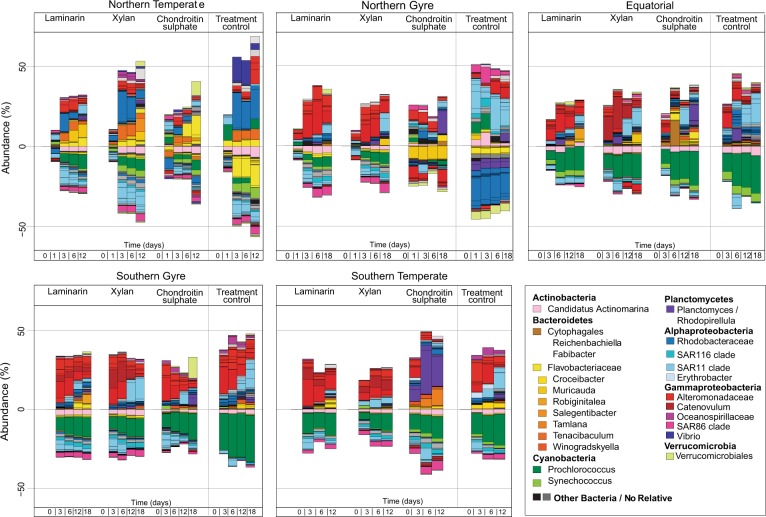
Substrate-related shift in the microbial communities during FLA-PS incubations. Bar charts show the percentage change in relative read abundance of individual bacterial genera. Incubations were carried out using three substrates (laminarin, xylan, chondroitin sulphate) and a treatment control (no substrate addition) and sampled at up to 5 time points between 0 and 18 days

**Fig. 5:**
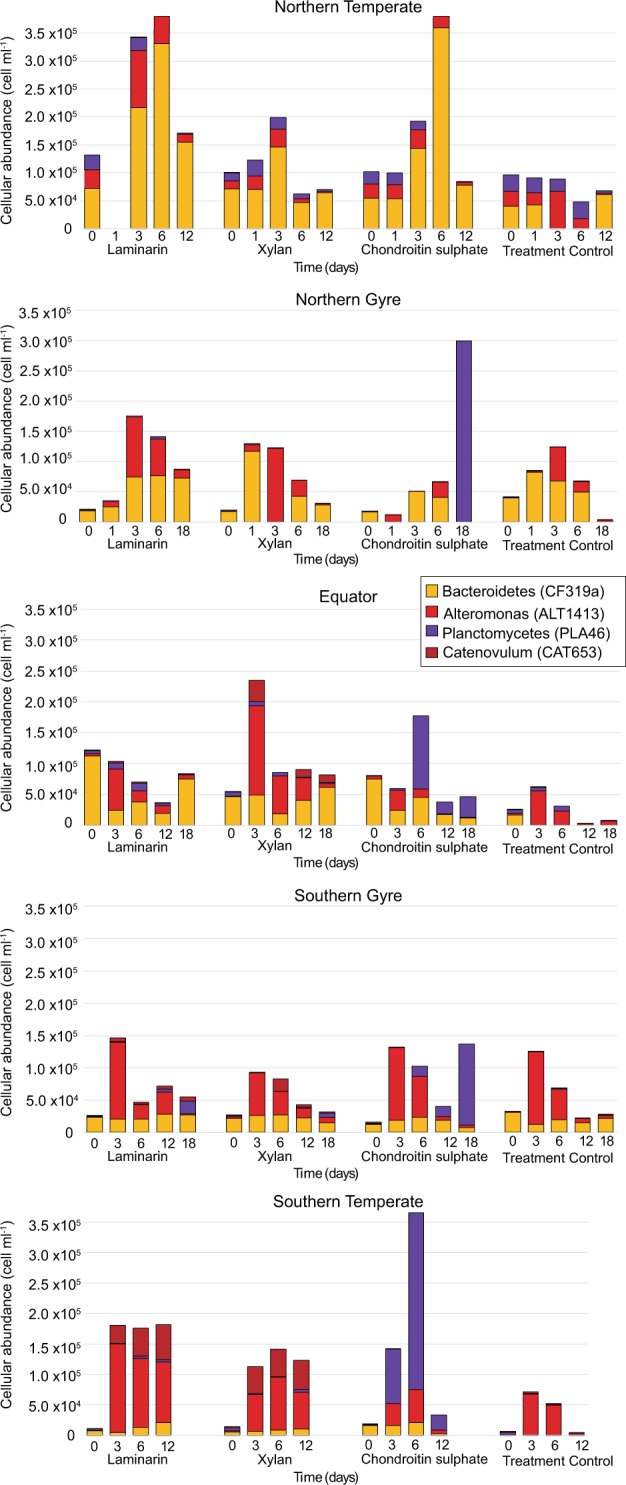
Absolute abundances of individual bacterial groups enumerated by FISH during FLA-PS incubations and in the treatment control. Group-specific FISH probes CF319a (orange), PLA46 (purple), CAT653 (dark red) and ALT1413 (red) target *Bacteroidetes*, *Planctomycetes*, *Catenovulum* and *Alteromonadaceae*, respectively. Panels: **a** Northern Temperate, **b** Northern Gyre, **c** Equatorial, **d** Southern Gyre and **e** Southern Temperate. Modified from [26]

Incubations with polysaccharides led to distinct substrate- and site-specific changes in microbial community composition. Statistical analyses of the change in community composition showed that during the incubations the bacterial communities varied significantly between substrates, over time and across stations (ANOSIM *R* = 0.07, *p* ≤ 0.001, *R* = 0.11, *p* ≤ 0.001, R = 0.44, *p* ≤ 0.001, respectively; Table 1 & Fig. 3). The highest amount of variance between the communities was explained by the sampling site (PERMANOVA *R*^2^ = 0.36, *p* = 0.001, Table 1), highlighting both the effect of the initial community composition and the similarity in the response of the communities to the individual substrates. Among all the stations, the community changes in the N.Temp station were most distinct. All incubations showed a relative and absolute increase in *Bacteroidetes* (7 × 10^4^ cells ml^−1^ to 3 × 10^5^ cells ml^−1^; Figs. 4 & 5), specifically the family *Flavobacteriaceae*, and the genera *Muricauda*, *Salegentibacter*, *Tenacibaculum* and uncultured *Flavobacteriaceae*. Additionally, there was an increase in the relative abundance of the alphaproteobacterial genus *Shimia* and the gammaproteobacterial genus *Colwellia*. The treatment control had a similar increase in *Shimia*, but also a marked increase in *Vibrio*, a change not seen in the substrate incubations.

**Table 1:** Permutational multivariant analysis of variance (PERMANOVA) and analysis of similarly (ANOSIM) of bacterial community composition based on Bray–Curtis dissimilarities of relative read abundance

	PERMANOVA				ANOSIM
Source of Variance	d.f.	SS	*pseudo* F	*R* ^2^	*R*
Substrate	2	1.19	12.6	0.04*	0.07*
Incubations time	6	4.45	15.64	0.16*	0.11*
Sampling site	4	10.24	54.04	0.36*	0.44*
Residuals	201	9.52		0.34	
Total	235	28.14		1	

	Pairwise PERMANOVA *p-*values		
Substrate	Chondroitin sulphate	Laminarin	Treatment contol
Laminarin	0.003	–	–
Treatment control	0.440	0.012	–
Xylan	0.003	0.094	0.015

Pairwise PERMANOVA (see above)

ANOSIM performed with 999 permutations

*d.f.* degrees of freedom, *SS* sum of squares

*p*-values reported between all three substrate incubations and the treatment control

*denotes significance of (*p* < 0.001) PERMANOVA test show influence of factors 'incubations time', 'substrate', 'sampling site' on community composition

*p*-values were obtained using sums of squares and 999 permutations

The most notable substrate-specific change was observed in the chondroitin sulphate incubations, where *Planctomycetes* increased nearly exclusively: at the N.Gyre and S.Gyre stations *Planctomycetes* increased after 12 to 18 days, and at the Equatorial and S.Temp stations after 3 to 6 days (8 × 10^2^ cells ml^−1^ to max: 3 × 10^5^ cells ml^−1^; Figs. 4, 5). The treatment controls, in contrast, showed no increase in the abundance of *Planctomycetes* (Fig. 5). In addition to the *Planctomycetes*, there was an increase in *Flavobacteriaceae* in the chondroitin sulphate incubations of the Equatorial and S.Temp stations, specifically the genera *Reichenbachiella* and *Tamlana* respectively. The S.Gyre station also showed an increase in *Verrucomicrobia* late in the chondroitin sulphate incubation (Fig. 4).

In the laminarin and xylan incubations at the N.Gyre, Equatorial, S.Gyre, and S.Temp stations, *Gammaproteobacteria* increased. In particular, *Catenovulum* grew from an undetectable abundance to 4 × 10^4^ cells ml^−1^ in the xylan and laminarin incubations of the Equatorial, S.Gyre, and S.Temp stations (Fig. 5); no *Catenovulum* were detected in the corresponding treatment controls (Fig. 5). Additionally, *Alteromonadaceae* increased in abundance in all substrate incubations of the N.Gyre, Equatorial, S.Gyre, and S.Temp stations after 3 days (1 × 10^3^ cells ml^−1^ to 1 × 10^5^ cells ml^−1^; Fig. 5). After this initial increase, there was a decrease in abundance from day 6 onwards at the N.Gyre, Equatorial, and S.Gyre stations, but not at the S.Temp station. The abundance of *Alteromonadaceae* also increased in the treatment controls of the N.Gyre, Equatorial, S.Gyre, and S.Temp stations, but only reached an average of 7 × 10^4^ cells ml^−1^ (Fig. 5).

In addition to increases in abundance, there were also decreases in relative abundance at all stations. These decreases occurred predominantly in the initially (T0) dominant groups of SAR86 and *Candidatus* Actinomarina, as well as *Prochlorococcus* and *Synechococcus*. The decrease in *Cyanobacteria* was doubtlessly because these phototrophs suffered from incubations in the dark. Similarly, SAR11 showed an initial decrease in abundance in all polysaccharide incubations, but increased again late (12–18 days) in the N.Gyre, Equatorial, and S.Gyre incubations.

The change in community composition of the treatment controls was significantly different from that of the laminarin and xylan incubations, but not of the chondroitin sulphate incubations (Table 1). The similarities between the treatment controls and the incubations could be due to the selection of similar groups by the naturally available DOM, to a bottle effect, or to the effects of grazing or viral lysis on the communities. However, the considerable relative change in abundance of specific groups in the polysaccharide incubations, accompanied by a moderate change in bulk abundance (Supplementary Figure S5), suggests a community-level response to substrate addition. Moreover, neither grazers nor viruses would be expected to respond directly to the specific nature of the added polysaccharide, so distinct responses to different polysaccharides (e.g., of *Planctomycetes* to chondroitin sulphate) suggest a dominantly substrate-driven microbial response.

### Identifying the substrate utilisation mechanisms of distinct bacterial groups

**Fig. 6:**
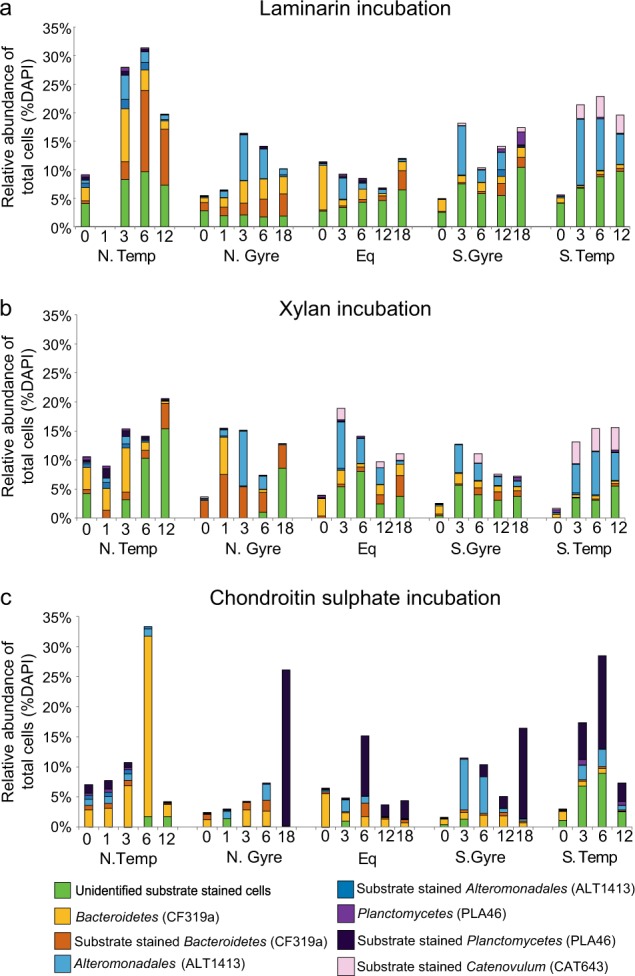
Relative abundance of FISH-identified and unidientified substrate-stained cells during FLA-PS incubations. Unidentified cells stained by individual substrates are shown in green. Relative abundance of *Bacteroidetes* (CF319a) and substrate stained *Bacteroidetes* are shown in yellow and orange. Relative abundance of *Alteromonadaceae* (ALT1413) and substrate stained *Alteromonadaceae* are shown in blue and dark blue. Relative abundance of *Planctomycetes* (PLA46) and substrate stained *Planctomycetes* are shown in purple and dark purple. Relative abundance of substrate stained *Catenovulum* (CAT653) are shown in pink; there were no unstained *Catenovulum*. Modified from [26]

The *Bacteroidetes*, *Catenovulum* and *Planctomycetes* showed substrate specific staining and could thereby be directly identified as ‘selfish’ bacteria (Fig. 6; [26]). However, although the *Bacteroidetes* showed direct uptake of substrate, their increase in abundance was not correlated with the increase in extracellular enzyme activities, suggesting that other organisms were also active.

In particular, the absolute and relative abundance of *Alteromonadaceae* was significantly correlated with the increase in hydrolysis rates in both the laminarin and the xylan incubations (0.41 ≥ R < 0.78, *p* < 0.05) Supplementary Figure S6). Although scavenging organisms would also increase in abundance as extracellular activity produces ‘public goods’, their dependency on the activity of the early responders should result in a growth delay. The direct correlation of absolute cell counts of *Alteromonadaceae* with measurements of extracellular enzymatic activity therefore suggests that they are external degraders.

Potential scavenging organisms were indirectly identified by analysing the latent changes in the microbial composition after hydrolysis rates peaked at day 3–6, when lower molecular weight hydrolysis products became more abundant in the incubations (as indicated by gel permeation chromatography of FLA-PS; Supplementary Figure S7). This trend was particularly apparent for the SAR11 clade, which decreased in relative abundance from day 1 to 6 and subsequently increased in relative abundance from day 12 to 18, especially at the N.Gyre, Equatorial, and S.Gyre stations (Fig. 4), a pattern observed predominantly in incubations where external degraders (*Alteromonadaceae*) had previously shown a high cell abundance. These data indicate possible scavenging of the ‘public goods’ produced by *Alteromonadaceae* or other sharing organisms.

## Discussion

Much of the organic matter remineralization in the ocean is carried out by heterotrophic microbial communities whose individual capabilities and group interactions are not yet fully understood. Identifying the roles played by different members of microbial communities in processing and respiring organic matter is crucial to understanding the complex ecological interactions within these communities, as well as the factors controlling carbon cycling on local and on broader scales.

During the AMT22 cruise, we serendipitously discovered that a substantial fraction of bacteria in the surface waters of the Atlantic Ocean use a ‘selfish’ mechanism to take up specific polysaccharides ([26] Fig. 1). Here, we link individual microorganisms to distinct substrate utilisation mechanisms by comparing selfish uptake to measurements of polysaccharide hydrolysis rates from the same incubations, and analysing microbial community dynamics in the polysaccharide incubations using NGS sequencing and FISH.

### Assigning substrate utilisation mechanisms

Determining the balance of substrate utilisation mechanisms used by microbial communities at different locations is complicated by the fact that multiple organisms act concurrently on individual components of organic matter in the ocean. Our use of structurally well-defined polysaccharides, added in comparatively low concentrations to water taken directly from the surface ocean, enables us to begin with a complex natural microbial community, and—via fluorescent tagging—specifically identify organisms carrying out selfish uptake of these polysaccharides. Additionally, the identities of bacteria that externally degrade polysaccharides and those acting as scavengers (Fig. 1) can then be inferred from changes in cellular and relative abundance, incorporating prior knowledge about the physiology of the respective bacterial groups.

Selfish organisms were identified as belonging to the *Bacteroidetes*, *Catenovulum* and *Planctomycetes* [26]. Marine *Bacteroidetes* are well known for their ability to use HMW substrates [10, 23, 47, 48], and members of the *Bacteroidetes* have been identified as carrying out ‘selfish’ uptake in the human gut [27]. However, beyond several well-studied members of the *Bacteroidetes* (e.g., [27, 49, 50]), the mechanisms and identities of organisms carrying out selfish uptake require further study.

For example, the two available genomes of the genus *Catenovulum* contain protein clusters annotated for xylan degradation (PCLA_890091: xylan 1,4-beta-xylosidase) as well as carbohydrate uptake (PRJNA200359: TonB family and susC/RagA family proteins), but their mechanism of selfish uptake remains to be determined [51–53]. Similarly, although the phylum *Planctomycetes* is well studied [54, 55], their means of selfish substrate uptake is also unknown. A recent study hints at the importance of pili-like fibres that originate from large crateriform structures on the cell surface, which could play a role in HMW substrate uptake [54].

Furthermore, although we could identify many of the selfish organisms using groups-specific FISH probes, a substantial fraction (on average 52%; median 45%) of the FLA-PS stained cells were not identified (Fig. 6). The capability to take up large fragments of polysaccharides thus is phylogenetically wide-spread, but a large fraction of the organisms that take up FLA-PS await identification.

Through the direct correlation of increases in abundance with the increase in activity the *Alteromonadaceae* were identified as external degraders. Previous studies have also found that *Alteromonadaceae*, specifically *Alteromonas*, increased significantly in abundance within hours of the addition of DOM or HMW DOM [22, 56]. Furthermore, omics-analysis of environmental samples [57] and individual isolates [58] have shown that *Alteromonas* have the metabolic capacity to degrade a diverse set of complex polysaccharides. Our results confirm this and suggest that *Alteromonas* are likely degrading polysaccharides predominantly using freely-released or cell-surface associated enzymes. The rapid increase in *Alteromonadaceae* at the N.Gyre, Equatorial, S.Gyre, and S.Temp stations supports the suggestion that *Alteromonadaceae* are r-strategists that can quickly make use of available substrates [57–60].

Other organisms doubtless also belong to the category of external degraders, given the fact that the ability to produce extracellular enzymes is phylogenetically widespread [17, 22, 61, 62]. In the absence of substrate staining, in fact, the distinction between selfish and sharing modes of substrate utilisation can only be made using focused biochemical and microbiological investigations of specific isolates (e.g., [27, 50]).

The timing of changes in relative abundance, in combination with genetic and physiological information, enabled us to identify indirectly potential scavengers. These organisms (e.g., the SAR11 clade) cannot actively degrade HWM substrates, but have a high abundance of low molecular weight transporters for both amino acids and sugars [63–66]. Although we did not quantify the absolute abundance of SAR11 by FISH, their relative abundance increased late in the incubations. The observed decrease in absolute abundance of other groups at earlier timepoints (day 3–6; Fig. 5) and the stability in TCC suggest that SAR11 grew using LMW hydrolysis products. Since SAR11 was not directly counted, however, it is also possible that the increase in relative abundance is due to grazing avoidance [67]. Additional organisms may also be functioning as scavengers; they may best be identified through targeted examination of genomes and isolate physiology [68].

### Linking patterns in activities to differences in microbial community composition

Comparison of activity patterns (hydrolysis rates and selfish uptake) and the changing composition of microbial communities during the incubations provides initial insights into factors controlling the rate and mode of polysaccharide degradation in distinct locations. One important consideration is that ‘selfish’ uptake, as discussed by Cuskin et al [27], is distinct from the extracellular hydrolysis measured in the incubations since these hydrolysis rates are calculated by analysing the change in the molecular weight distribution of the FLA-PS remaining after 0.2 µm filtration. Any labelled bacteria are retained on the filter and therefore removed from the filtrate; the FLA-PS that these cells retain is therefore likewise removed. The hydrolysis rates (Fig. 2a) and the abundance of labelled cells (Fig. 2b) thus indicate two potentially distinct processes: on the one hand, enzymatic hydrolysis occurring outside the cell—in which the hydrolysate is not immediately passed into the periplasm—and on the other, uptake of large pieces of polysaccharide without much loss to the external environment (Fig. 1). However, this view of strictly-selfish behaviour may be oversimplified when applied to complex natural microbial communities. Some selfish organisms may also actively or passively produce hydrolysis products, as has been found for specific members of the gut microbiota [50]. The relationship between the abundance of FLA-PS labelled cells, and extracellular hydrolysis rates thus is complex (Fig. 2).

**Fig. 7:**
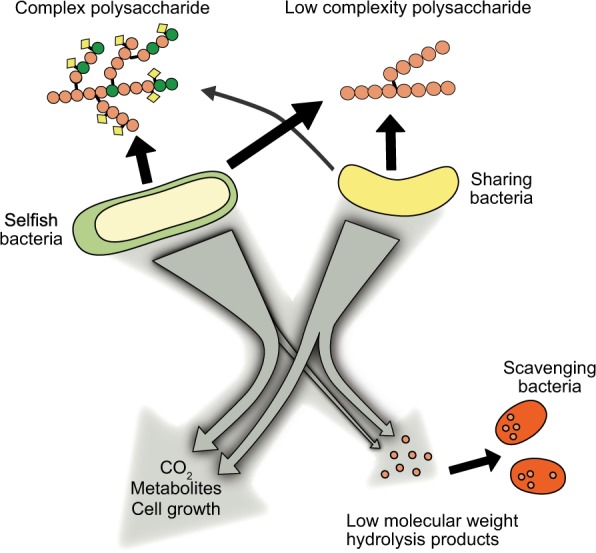
Conceptional figure showing factors affecting the pathways of HMW substrates processing. The production of LMW products is dependent on the relative balance (growth rate and population size) and relative activities of selfish and sharing organisms. A high flux of extracellular hydrolysis supports a larger scavenging community, whereas predominantly selfish uptake leads to fewer scavenging organisms. Additionally, the level of relative activity is dependent on the complexity of the substrate. Arrow sizes represents relative proportions

One additional variable that likely plays a role in determining the balance between selfish uptake and extracellular hydrolysis is the complexity of the enzymatic system required for degradation of a specific substrate. Algal polysaccharides can be highly complex, and bacteria that metabolise these polysaccharides must produce a large number of enzymes, binding proteins, and transporters [2, 69] likely favouring selfish uptake (Fig. 7). Consequently, resource partitioning and niche differentiation characterise natural bacterial communities that degrade specific algal polysaccharides [12, 21]. The degradation of structurally-less complex polysaccharides, however, may be carried out by a wider range of organisms that could act in a functionally redundant manner. Of the substrates we tested, laminarin and xylan belong to this category and were utilised readily by both selfish and sharing bacteria (Fig. 7).

Compared to laminarin and xylan, other substrates - such as chondroitin sulphate—clearly were targeted by a much narrower range of bacteria. In the chondroitin sulphate incubations of all except the N.Temp station, there was a substrate-specific selection of *Planctomycetes*, specifically the genera *Rhodopirellula* and *Planctomyces*. Aside from *Bacteroidetes* in the N.Temp station, no other organisms appeared to take up chondroitin sulphate. Moreover, hydrolysis rates of chondroitin sulphate were spatially and temporally highly variable, and in general, were detected only after there was an increase in *Planctomycetes*. This may be a case where selfish uptake nonetheless yielded some public goods (i.e., fragments of hydrolysed polysaccharides were released), or an instance of ‘sensing’ enzymes [70] producing externally-released hydrolysate. In any case, both the *Planctomycetes* and *Bacteroidetes* contain sulfatases that would enable them to degrade chondroitin sulphate [48, 69, 71, 72], and representative isolates do in fact grow on chondroitin sulphate [72, 73].

Other substrates were also targeted only by a narrow range of bacteria. The observation that fucoidan uptake was measurable at low levels, but hydrolysis was not, suggests that this substrate can be taken up in a selfish manner, and also is biochemically more difficult to process, a hypothesis supported by data demonstrating that it is hydrolysed by highly complex enzyme systems [74]. Although samples from the arabinogalactan and pullulan incubations were not sequenced or subject to FISH analysis, extracellular hydrolysis of these substrates was detected only at time points well after a considerable fraction of cells were stained with these specific FLA-PS (Fig. 2), suggesting that the ability to enzymatically target these substrates also may not be widespread among marine bacteria. Investigations to date demonstrate that hydrolysis of arabinogalactan, fucoidan, and pullulan is indeed spatially quite patchy in surface ocean waters [7, 75, 76].

An additional factor determining the balance between selfish uptake and extracellular hydrolysis is the composition of the initial bacterial community. In fact, our study shows that differences in the community composition between stations had a pronounced effect on the hydrolysis patterns and the mode of substrate utilisation. For example, at the S.Gyre station, the extracellular hydrolysis rates of laminarin and xylan were very high, but there was a very low level of selfish activity (Fig. 2). The rapid hydrolysis of polysaccharides by external degraders—likely members of the *Alteromonadaceae*, which were comparatively abundant in the incubations (Fig. 4)—would decrease the availability of the polysaccharides and could result in a limited growth of selfish organisms; the external degraders, in this case, out-compete selfish organisms. We observed this trend in both the Equatorial and the S.Gyre stations, where laminarin and xylan were rapidly hydrolysed. A notable observation from these incubations is that although the external degraders had a significant effect on the substrate hydrolysis, their initial abundance was very low (Fig. 5). Therefore, organisms with a low initial abundance, but high growth rate can have a disproportionally large effect on organic matter turnover [60].

At the N.Temp station there was a pronounced difference in the initial population structure compared to other stations; specifically, a higher abundance of *Flavobacteriaceae* and a lower abundance of *Alpha-* and *Gammaproteobacteria*. At this station, selfish organisms appeared to out-compete external degraders: after 5 min, five of the six substrates showed selfish activity (Fig. 2). In the laminarin, xylan, and chondroitin sulphate incubations, this activity could be directly related to specific genera of the *Flavobacteriaceae* (Supplementary Figure S8), which increased significantly in abundance during the incubations (Fig. 5). Their higher initial abundance may have resulted in a competitive advantage at the initial phase of substrate addition, enabling them to out-compete external degraders.

### Multiple modes of substrate utilisation: consequences for carbon cycling

The observation that the same substrate can be processed in different ways by different members of pelagic microbial communities points to significant follow-on effects for carbon cycling in the ocean. Whether or not LMW hydrolysis products are made available to scavenging organisms depends on the relative balance and relative activities of selfish and sharing organisms (Fig. 7). A high flux of extracellular hydrolysate may support a larger community of scavengers, whereas a predominantly selfish mode of uptake may lead to fewer scavenging organisms; therefore, the same quantity of a given substrate could have profoundly different effects. These results are also highly relevant for consideration of microbial energetics: on the cellular level, the investment in more complex polysaccharide-degrading machinery provides the opportunity to utilise substrates that are otherwise unavailable (Fig. 7). Such an investment may pay off because selfish uptake secures all of the substrate for the cell, without loss to the external environment.

Likewise, the prevalence of selfish substrate uptake in the ocean requires reconsideration of models that have been used to assess the ‘economic return’ on production of extracellular enzymes. The hydrolysis of HMW substrates has typically been considered a high-cost aspect of microbially-driven carbon cycling: an investment of carbon, nitrogen, and energy is required to produce extracellular enzymes [29, 77]. The extent to which this investment pays off for an organism is directly related to the return obtained by the producing cell, a return calculated as the hydrolysate that is captured [28, 78]. These models also account for substrate loss, via diffusion and especially via capture by other organisms (cheaters/scavengers) that have not invested in the production of enzymes. Recalibration of these two-player models is now necessary. At a minimum, a three-player model would be more appropriate, with selfish, sharing, and scavenging organisms all responsible for some fraction of organic matter transformation and respiration (Fig. 1). Such a model, however, should also take into consideration the complexity of the enzyme system required for hydrolysis of a given substrate, rather than modelling all substrates as requiring the same enzymatic effort. Our results suggest that at a minimum, a high and low complexity investment should be considered (Fig. 7). Precise quantification of the substrates that are processed via different mechanisms, and of the conditions under which different mechanisms predominate, will require follow-up experiments that can be designed with prior knowledge of the existence of these different modes of substrate utilisation [79–83].

## Supplementary information

Supplementary Figure Legends

Supplementary Methods

Supplementary Table S1

Supplementary Figure S1

Supplementary Figure S2

Supplementary Figure S3

Supplementary Figure S4

Supplementary Figure S5

Supplementary Figure S6

Supplementary Figure S7

Supplementary Figure S8

Supplementary Figure S9

Supplementary Figure S10
