# Supplementary material for: Selfish, sharing and scavenging bacteria in the Atlantic Ocean: a biogeographical study of bacterial substrate utilisation
Source: ISME J. 2018 Dec 7;13(5):1119–32. doi: 10.1038/s41396-018-0326-3 (PMC6474216; doi:10.1038/s41396-018-0326-3)
Supplement: Supplementary file 1 — Supplementary Figure Legends [file 41396_2018_326_MOESM1_ESM.docx]

Supplementary Figure Legends

Supplementary Figure S1: Sampling sites in the Atlantic Ocean. (a) Sites shown by white dots; background colours indicate the average chlorophyll a concentration (mg m) during the sampling (map obtained from MODIS, Ocean Biology Processing Group (2014). (b) Latitude and longitude of each sampling site. Figure from Reintjes et al. 2017

Supplementary Figure S2: Bubble plot of bacterial genera with a minimum relative read abundance of 0.5% in all initial (T0) samples of the N. Temperate, N. Gyre, Equatorial, S. Gyre and S.Temperate stations (depicted by green boxes). The size of the bubbles indicates the average relative abundance (%) of each genus. The dominant genera are highlighted in bold font.

Supplementary Figure S3: NMDS plot showing Bray Curtis dissimilarity between the initial (T0) bacterial community composition at each station (N. Temperate, N. Gyre, Equatorial, S. Gyre and S. Temperate) along the AMT22. ANOSIM analysis shows a significant difference between the stations (R = 0.48, *p* = >0.001).

Supplementary Figure S4: Average relative read abundance of bacterial genera in all initial (T0) samples of the N. Temperate, N. Gyre, Equatorial, S. Gyre and S. Temperate stations. Bar colors show each genera's phylogenetic affiliation *Bacteroidetes* (yellow), *Cyanobacteria* (green), *Alphaproteobacteria* (blue), *Gammaproteobacteria* (purple) and other Bacteria (red).

Supplementary Figure S5: Change in absolute cellular abundance (cell ml) during each substrate incubation (laminarin, xylan, chondroitin) and unamended treatment control over time in the N. Temperate, N. Gyre, Equatorial, S. Gyre, and S. Temperate station. The error bars indicate the total range of triplicate incubations. Modified from Reintjes et al., 2017

Supplementary Figure S6: Spearman's Rank-Order correlations between the substrate hydrolysis rate (laminarin, xylan and chondroitin sulphate) and change in absolute abundance (groups specific FISH counts) and relative abundance (normalised read abundance). Positive correlations are displayed in blue and negative in correlations are displayed in red. The colour intensity and size of the circle are proportional to the correlation coefficients, displayed to the right of the circle. The legend colours on the right show the correlation coefficient and the corresponding colour. Insignificant correlations are left blank. Correlations with *p*-values >0.05 were considered insignificant.

Supplementary Figure S7. Change in the molecular weight distribution of the total added fluorescently labelled laminarin pool over time in incubations from the Northern Temperate station. Measurements were done using gel permeation chromatography.

Supplementary Figure S8: Barchart of the relative read abundance of *Bacteroidetes* genera in all initial (T0) samples of the N. Temperate, N. Gyre, Equatorial, S. Gyre and S. Temperate station. Genera are color coded and sorted by taxonomy.

Supplementary Figure S9: a) Change in absolute cellular abundance (cell ml) during substrate incubations (laminarin, xylan, chondroitin sulphate) and unamended treatment control in the S. Gyre. Error bars indicate the total range of triplicates. b) Barchart showing the bacterial community composition within biological triplicate substrate incubations and treatment control (not in triplicate) of the S. Gyre. All incubations were sampled at 0, 3, 6, 12 and 18 days.

Supplementary Figure S10: Standard deviation (sd) and 95% confidence intervals (error bars) of the relative read abundance within biological triplicates. The minimum and maximum standard deviations of all triplicates are indicated by blue dots. Samples with a standard deviation within the 95% confidence interval are highlighted in orange.
