# Supplementary material for: Selfish, sharing and scavenging bacteria in the Atlantic Ocean: a biogeographical study of bacterial substrate utilisation
Source: ISME J. 2018 Dec 7;13(5):1119–32. doi: 10.1038/s41396-018-0326-3 (PMC6474216; doi:10.1038/s41396-018-0326-3)
Supplement: Supplementary file 2 — Supplementary Methods [file 41396_2018_326_MOESM2_ESM.docx]

Supplementary Methods

Microbial Diversity Analysis

DNA Extraction

Microbial DNA was extracted from all biological triplicates of the laminarin, xylan and chondroitin sulphate incubations, as well as the corresponding treatment controls, using the MoBio Power Water DNA Extraction Kit (MoBio Laboratories, Inc) as recommended by the manufacturer.

PCR and Sequencing

PCR was carried out using the primers S-D-Bact-0341-b-S-17 (5′-CCTACGGG NGGCWGCAG-3′) and S-D-Bact-0785-a-A-21 (5′-GACTACHVGGGTATCTAATCC-3) targeting the V3-V4 variable region of the 16S rRNA (Klindworth et al 2013) in combination with Phusion High-Fidelity DNA polymerase (Thermo Fisher). Subsequently, the PCR products were visualised, and amplicon bands were cut out with a sterile scalpel. The excised gel slices were purified using the QiagenMiniElute kit (Qiagen). After purification, the PCR products were pooled into libraries with a minimum DNA concentration of 1 µg, measured using a Qubit assay (Invitrogen). The libraries were paired-end sequenced using the Miseq Reagents Kit V3 for 2 x 300 bp sequencing on an Illumina Miseq (Illumina, CA, USA) at the Max Planck Genome Centre (Cologne, Germany)

Processing of Sequencing Data

The sequences were visually quality checked using FASTQC (Andrews 2010). Subsequently, the reads were merged and quality trimmed using BBMerge (JGI, CA, USA) with the settings qtrim=T, trimq=20, minlength=290, maxlength=550, minoverlap=60, strict=T. The trimmed, merged reads were further quality trimmed, filtered by length and demultiplexed using Mothur (Schloss et al 2009), with the command trim.seqs (minlength=350, maxambig=0, maxhop=8).

The quality trimmed merged reads were then processed using the recommended settings of the bioinformatics pipeline of the SILVAngs project (Quast et al 2013). Briefly, processing involved alignment against the SSU rRNA seed of the SILVA database release 119 using SINA v1.2.10 (Quast et al 2013) and subsequent quality controls for sequence length (> 200 bp), minimum quality score (30), minimum alignment score (40), minimum alignment identify (40%), maximum ambiguities (< 2%) and maximum homopolymers (< 2%). The remaining reads were then de-replicated, clustered and classified. De-replication and clustering was done using cd-hit-est (version3.1.2; Li and Godzik, 2006), running in accurate mode, ignoring overhangs and applying identity criteria of 1.00 and 0.98, respectively. The classification was performed by a local nucleotide BLAST search against SILVA SSURef 119.1 NR database using blast -2.2.22+ with standard settings. A detailed description of the SILVAngs project and pipeline can be found (<https://www.arb-silva.de/ngs/Index.html#about>: Quast et al 2013).

All raw and merged sequence data was deposited in the European Nucleotide Archive (ENA, (Silvester 2018)) using the data brokerage service of the German Federation for Biological Data, (GFBio, (Diepenbroek et al 2014)), in compliance with the MIxS standard (Yilmaz et al 2011). The INSDC accession number for the data is: PRJEB28155.

Evaluation of Sequencing Triplicates

Each triplicate of the laminarin, xylan and chondroitin sulphate incubation, as well as the corresponding treatment control, was sequenced at each timepoint. The bacterial community composition and total cellular abundance at a given time point within individual triplicates was highly similar and not significantly different for 79% of all samples (Supplementary Figure S9 & S10). Furthermore, the triplicate incubations showed a similar change in diversity and abundance over time (Supplementary Figure S9). The similarity among triplicates was calculated by first obtaining the mean variance of the triplicate read abundance from each genus and then calculating the mean variance in each sample (all genera). The standard deviation was calculated by taking the square root of the mean variance of each sample. Subsequently, the confidence intervals were calculated. The mean variance of all triplicate incubations was 1.62 x 10^-5^ with a mean standard deviation of 3.6 x 10^-3^ and 95% confidence interval of 2.37 x 10^-4^; 79% of the samples fell within the confidence intervals (Supplementary Figure S10).

Microbial Diversity Analysis and Statistics

The change in community composition over the course of each incubation was investigated using the percentage change in abundance of each genus over time (minimum read abundance of 0.5%). Percentage change in abundance is calculated by analysing the change in normalised read abundance of each bacterial genus over time compared to the initial community (T0). This calculation highlighted both the positive and negative responses of each genus to the substrate addition.

The interpretation and visualisation of the microbial diversity data was done using normalised genus abundance to site matrices in the R software with the packages Vegan (community ecology package (Oksanen et al 2013)) and Rioja (Analysis of Quaternary Science Data (Juggins, 2016). Normalisation was done using the decostand(method = “total”) function of the Vegan software package. After normalisation, Bray-Curtis dissimilarity matrices of the bacterial community of all samples (triplicates and treatment control) were constructed.

Beta diversity analysis, community ordination, correlations and statistical analysis (analysis of similarity (ANOSIM), permutation multivariant analysis of variance (PERMANOVA) and pairwise PERMANOVA) were performed in R using the packages Vegan, Corrplot, and Rojia (Oksanen et al 2013, Juggins,2016, Wei and Simon 2017).

Initial differences in the community structure between sampling sites were analysed by comparing the T0 samples (triplicates and treatment control) by ANOSIM and visualised in non-metric multi-dimensional scaling plots (NMDS). Substrate specific changes in the community composition, as well as between incubations and treatment controls were analysed using ANOSIM and visualised using NMDS plots. PERMANOVA was performed to identify the amount of variance associated with individual factors (substrate, sampling site and time) and pairwise PERMANOVA were performed to compare between groups.

Spearman’s rank order correlations between enzyme activities and the change in absolute abundance (FISH counts) and relative abundance (NGS sequencing) within each incubation (triplicate) were performed to identify the extracellularly active organisms.
