# Supplementary material for: Selfish, sharing and scavenging bacteria in the Atlantic Ocean: a biogeographical study of bacterial substrate utilisation
Source: ISME J. 2018 Dec 7;13(5):1119–32. doi: 10.1038/s41396-018-0326-3 (PMC6474216; doi:10.1038/s41396-018-0326-3)
Supplement: Supplementary file 3 — Supplementary Table S1 [file 41396_2018_326_MOESM3_ESM.docx]

Supplementary Table S1 | FISH probes used in this study (Modified from Reintjes et al., 2017)

| Probe Name | Sequence 5' - 3' | FA% | Reference |
| --- | --- | --- | --- |
| CF319a  PLA46  CAT653  ALT1413 | TGGTCCGTGTCTCAGTAC  GACTTGCATGCCTAATCC  CCCCCTCTCCCTTACTCT  TTTGCATCCCACTCCCAT | 35  30  25  40 | Manz *et al.,*1992  Neef et al., 1998  Reintjes et al., 2017  Pernthaler et al., 2000 |

**FA corresponds to the formamide concentration using in the hybridisation buffer.*
