# Supplementary material for: Selfish, sharing and scavenging bacteria in the Atlantic Ocean: a biogeographical study of bacterial substrate utilisation
Source: ISME J. 2018 Dec 7;13(5):1119–32. doi: 10.1038/s41396-018-0326-3 (PMC6474216; doi:10.1038/s41396-018-0326-3)
Supplement: Supplementary file 4 — Supplementary Figure S1 [file 41396_2018_326_MOESM4_ESM.pdf]

a

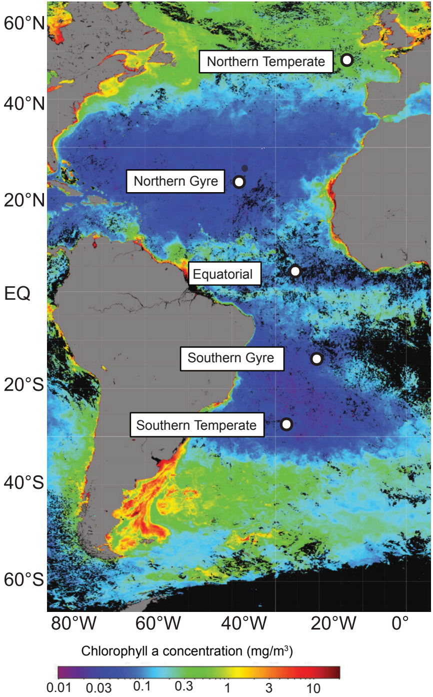

b

Site	Latitude	Longitude
Northern Temperate	41°39N	21°15W
Northern Gyre	22°04N	39°47W
Equatorial	2°55N	25°39W
Southern Gyre	11°37S	25°10W
Southern Temperate	26°57S	25°00W

Supplementary Figure S1: Sampling sites in the Atlantic Ocean. (a) Sites shown by white dots; background colours indicate the average chlorophyll a concentration (mg m^{-3}) during the sampling (map obtained from MODIS, Ocean Biology Processing Group (2014). (b) Latitude and longitude of each sampling site. Figure from Reintjes et al. 2017
