# Supplementary material for: Selfish, sharing and scavenging bacteria in the Atlantic Ocean: a biogeographical study of bacterial substrate utilisation
Source: ISME J. 2018 Dec 7;13(5):1119–32. doi: 10.1038/s41396-018-0326-3 (PMC6474216; doi:10.1038/s41396-018-0326-3)
Supplement: Supplementary file 5 — Supplementary Figure S2 [file 41396_2018_326_MOESM5_ESM.pdf]

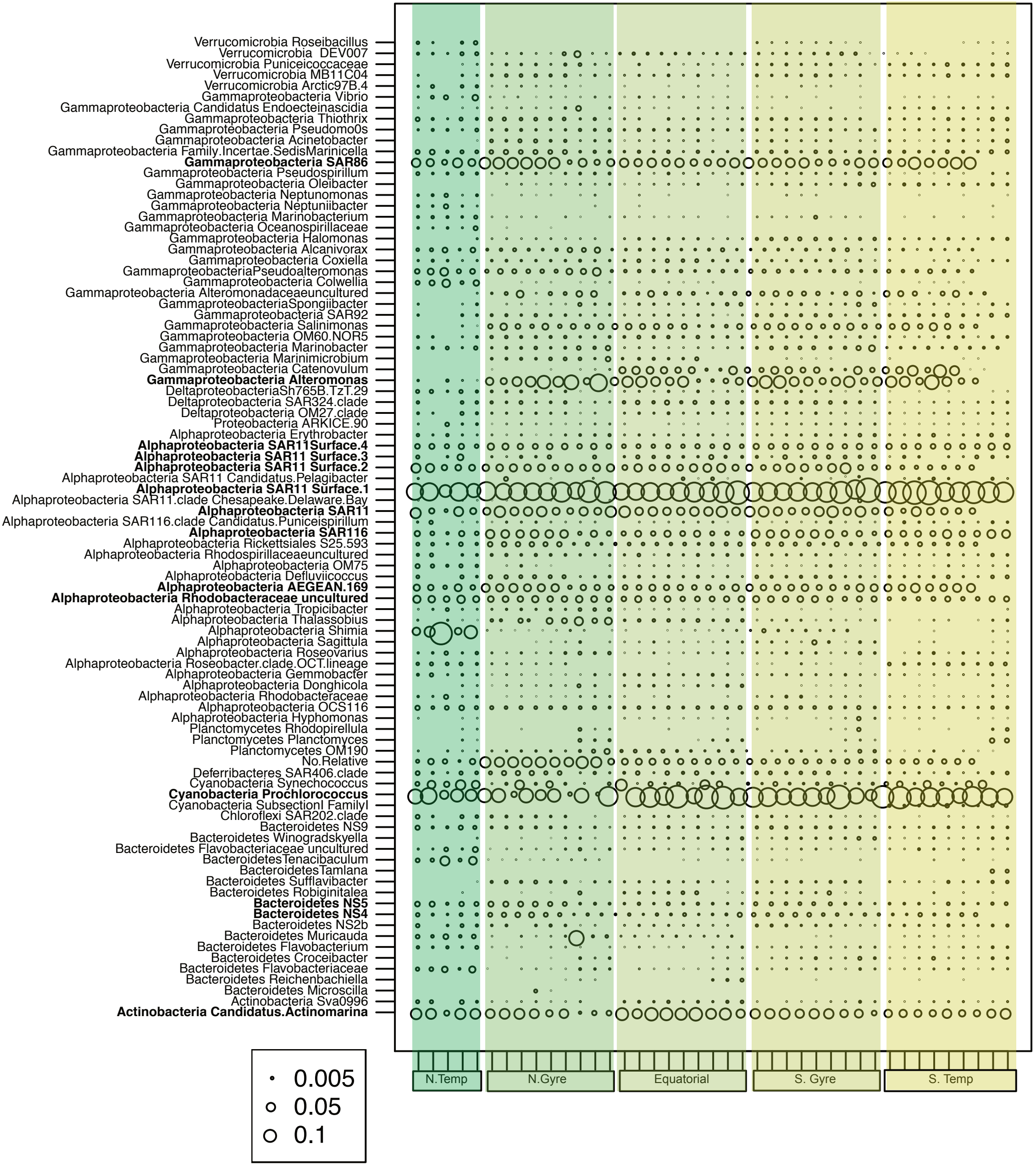


Supplementary Figure S2: Bubble plot of bacterial genera with a minimum relative read abundance of 0.5% in all initial (T0) samples of the N. Temperate, N. Gyre, Equatorial, S. Gyre and S.Temperate stations (depicted by green boxes). The size of the bubbles indicates the average relative abundance (%) of each genus. The dominant genera are highlighted in bold font.
