# Supplementary material for: Selfish, sharing and scavenging bacteria in the Atlantic Ocean: a biogeographical study of bacterial substrate utilisation
Source: ISME J. 2018 Dec 7;13(5):1119–32. doi: 10.1038/s41396-018-0326-3 (PMC6474216; doi:10.1038/s41396-018-0326-3)
Supplement: Supplementary file 6 — Supplementary Figure S3 [file 41396_2018_326_MOESM6_ESM.pdf]

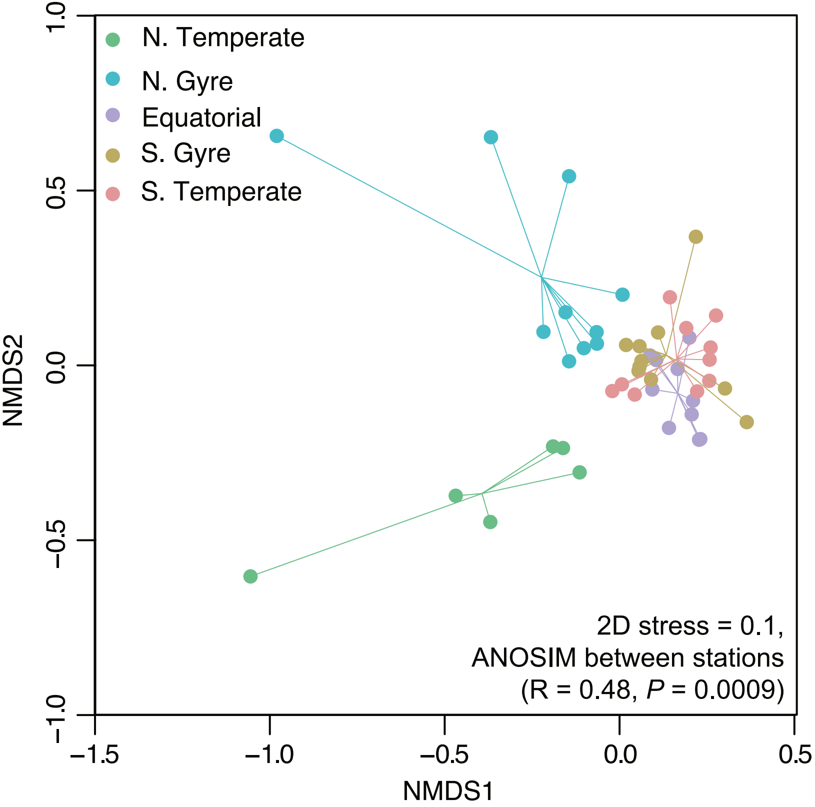

Supplementary Figure S3: NMDS plot showing Bray Curtis dissimilarity between the initial (T0) bacterial community composition at each station (N. Temperate, N. Gyre, Equatorial, S. Gyre and S. Temperate) along the AMT22. ANOSIM analysis shows a significant difference between the stations ($R = 0.48$, $P = 0.0009$).
