# Supplementary material for: Selfish, sharing and scavenging bacteria in the Atlantic Ocean: a biogeographical study of bacterial substrate utilisation
Source: ISME J. 2018 Dec 7;13(5):1119–32. doi: 10.1038/s41396-018-0326-3 (PMC6474216; doi:10.1038/s41396-018-0326-3)
Supplement: Supplementary file 7 — Supplementary Figure S4 [file 41396_2018_326_MOESM7_ESM.pdf]

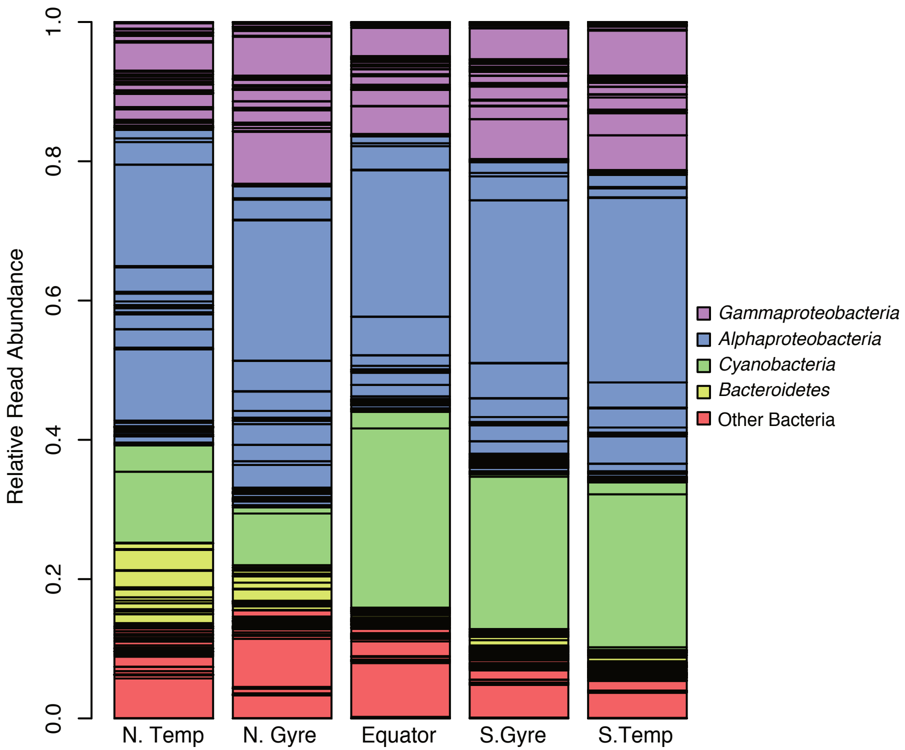

Supplementary Figure S4: Average relative read abundance of bacterial genera in all initial (T0) samples of the N. Temperate, N. Gyre, Equatorial, S. Gyre and S. Temperate stations. Bar colors show each genera's phylogenetic affiliation *Bacteroidetes* (yellow), *Cyanobacteria* (green), *Alphaproteobacteria* (blue), *Gammaproteobacteria* (purple) and other Bacteria (red).
