# Supplementary material for: Selfish, sharing and scavenging bacteria in the Atlantic Ocean: a biogeographical study of bacterial substrate utilisation
Source: ISME J. 2018 Dec 7;13(5):1119–32. doi: 10.1038/s41396-018-0326-3 (PMC6474216; doi:10.1038/s41396-018-0326-3)
Supplement: Supplementary file 8 — Supplementary Figure S5 [file 41396_2018_326_MOESM8_ESM.pdf]

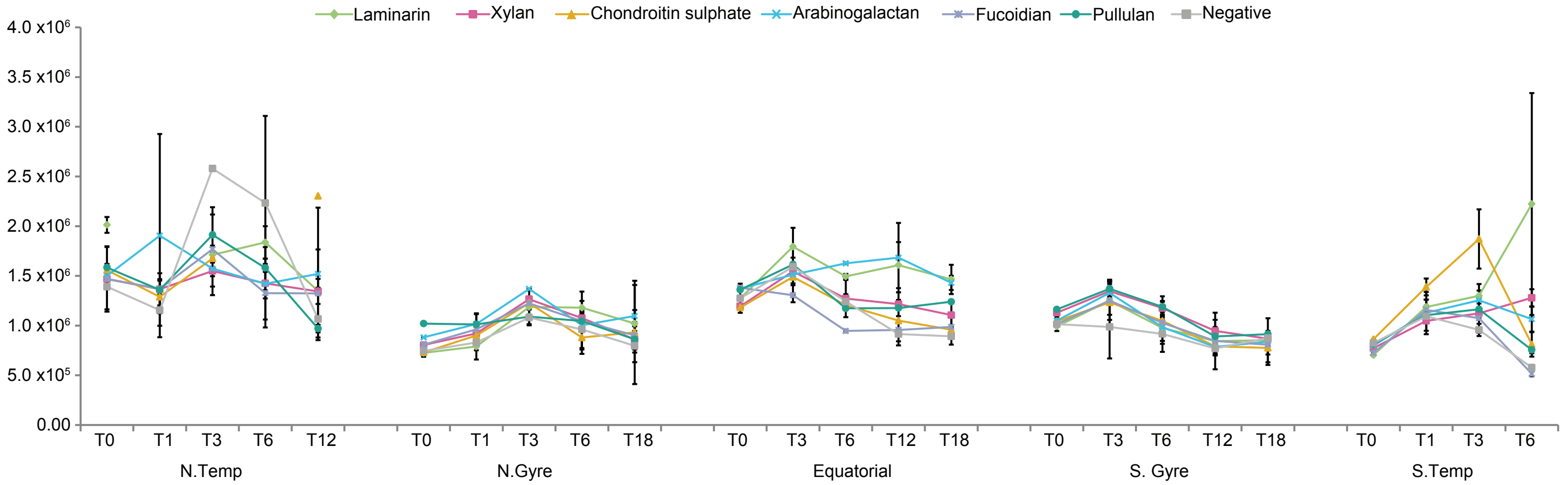

Supplementary Figure S5: Change in absolute cellular abundance (cell ml^{-1}) during each substrate incubation (laminarin, xylan, chondroitin) and unamended treatment control over time in the N. Temperate, N. Gyre, Equatorial, S. Gyre, and S. Temperate station. The error bars indicate the total range of triplicate incubations. Modified from Reintjes et al., 2017
