# Supplementary material for: Selfish, sharing and scavenging bacteria in the Atlantic Ocean: a biogeographical study of bacterial substrate utilisation
Source: ISME J. 2018 Dec 7;13(5):1119–32. doi: 10.1038/s41396-018-0326-3 (PMC6474216; doi:10.1038/s41396-018-0326-3)
Supplement: Supplementary file 9 — Supplementary Figure S6 [file 41396_2018_326_MOESM9_ESM.pdf]

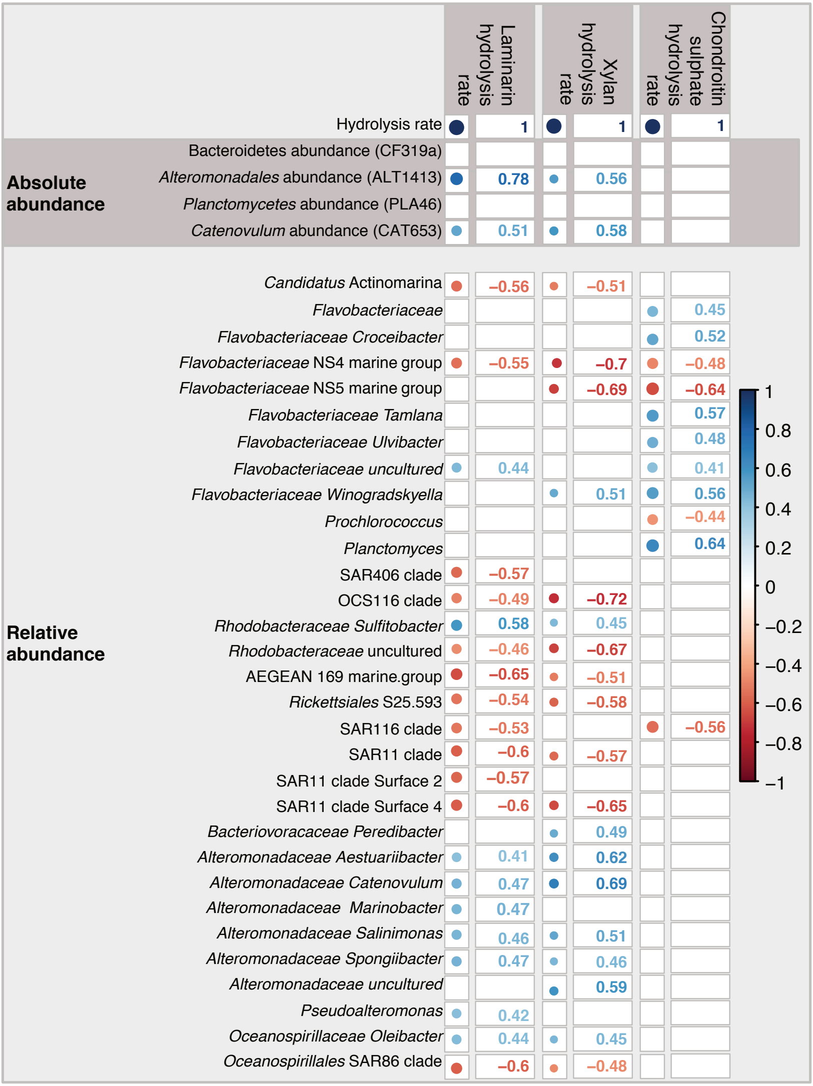

Supplementary Figure S6: Spearman's Rank-Order correlations between the substrate hydrolysis rate (laminarin, xylan and chondroitin sulphate) and change in absolute abundance (groups specific FISH counts) and relative abundance (normalised read abundance). Positive correlations are displayed in blue and negative in correlations are displayed in red. The colour intensity and size of the circle are proportional to the correlation coefficients, displayed to the right of the circle. The legend colours on the right show the correlation coefficient and the corresponding colour. Insignificant correlations are left blank. Correlations with p-values >0.05 were considered insignificant.
