# Supplementary material for: Selfish, sharing and scavenging bacteria in the Atlantic Ocean: a biogeographical study of bacterial substrate utilisation
Source: ISME J. 2018 Dec 7;13(5):1119–32. doi: 10.1038/s41396-018-0326-3 (PMC6474216; doi:10.1038/s41396-018-0326-3)
Supplement: Supplementary file 10 — Supplementary Figure S7 [file 41396_2018_326_MOESM10_ESM.pdf]

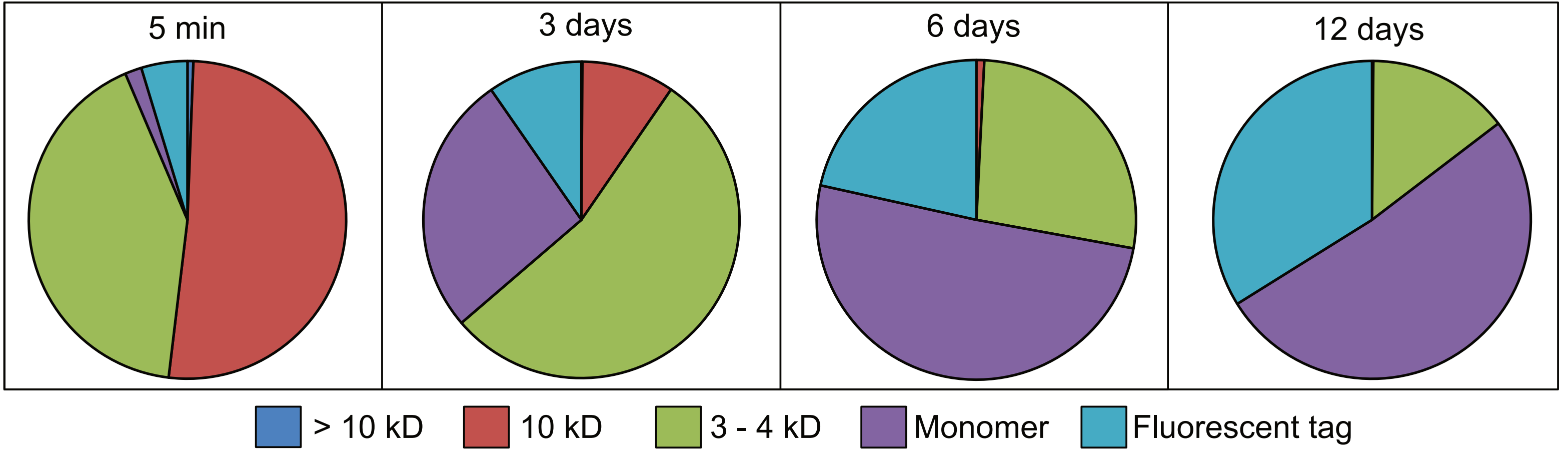

Supplementary Figure S7: Change in the molecular weight distribution of the total added fluorescently labelled laminarin pool over time in incubations from the Northern Temperate station. Measurements were done using gel permeation chromatography.
