# Supplementary material for: Selfish, sharing and scavenging bacteria in the Atlantic Ocean: a biogeographical study of bacterial substrate utilisation
Source: ISME J. 2018 Dec 7;13(5):1119–32. doi: 10.1038/s41396-018-0326-3 (PMC6474216; doi:10.1038/s41396-018-0326-3)
Supplement: Supplementary file 11 — Supplementary Figure S8 [file 41396_2018_326_MOESM11_ESM.pdf]

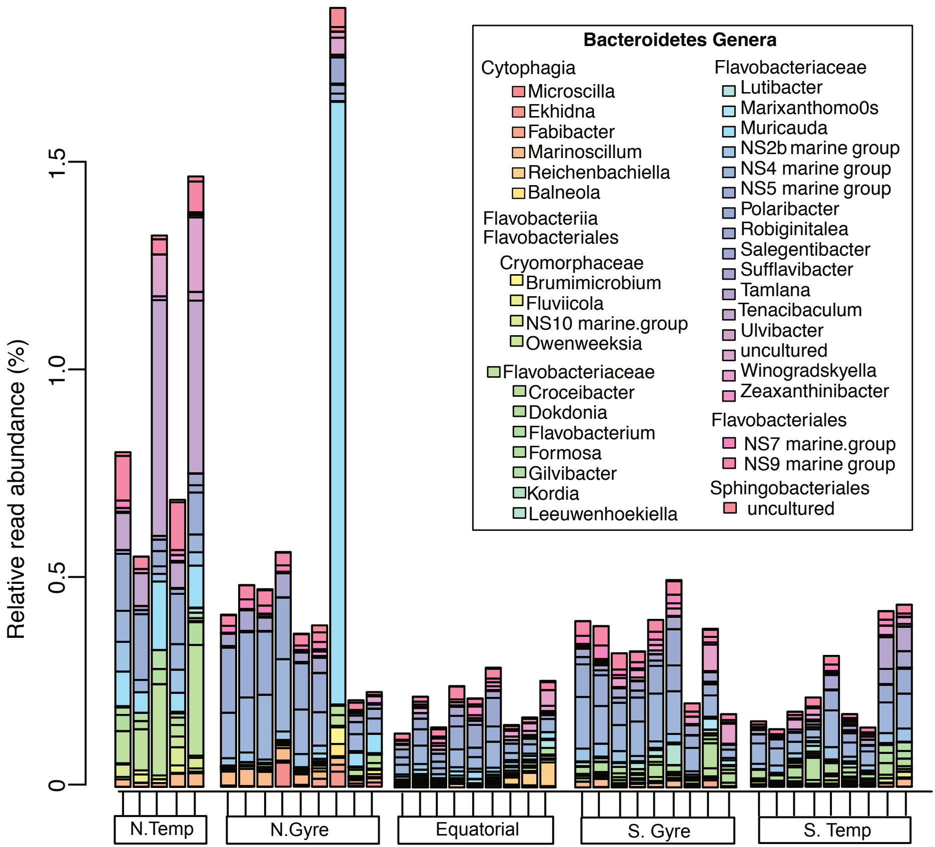

Supplementary Figure S8: Barchart of the relative read abundance of *Bacteroidetes* genera in all initial (T0) samples of the N. Temperate, N. Gyre, Equatorial, S. Gyre and S. Temperate station. Genera are color coded and sorted by taxonomy.
