# Supplementary material for: Selfish, sharing and scavenging bacteria in the Atlantic Ocean: a biogeographical study of bacterial substrate utilisation
Source: ISME J. 2018 Dec 7;13(5):1119–32. doi: 10.1038/s41396-018-0326-3 (PMC6474216; doi:10.1038/s41396-018-0326-3)
Supplement: Supplementary file 12 — Supplementary Figure S9 [file 41396_2018_326_MOESM12_ESM.pdf]

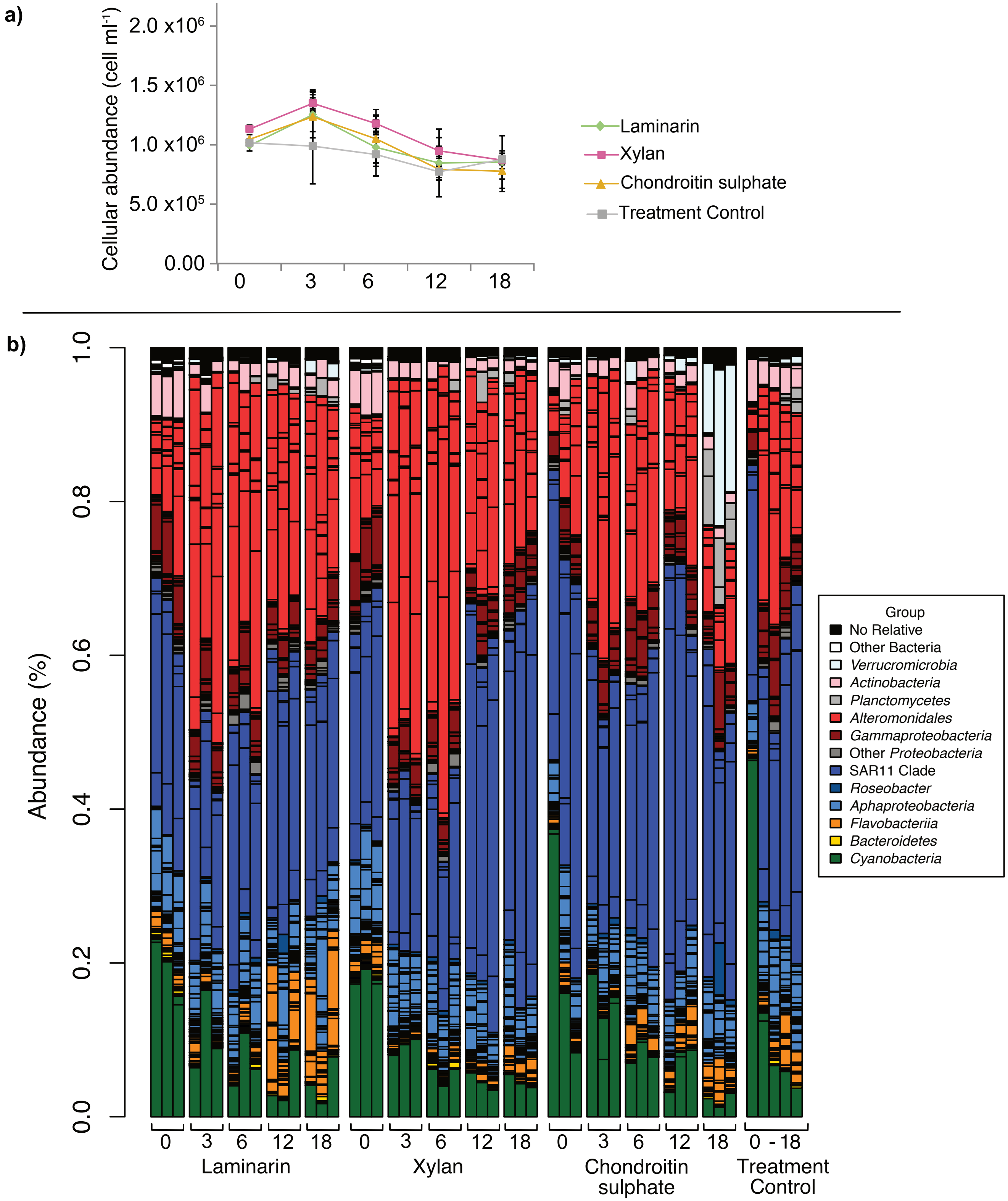

Supplementary Figure S9: a) Change in absolute cellular abundance (cell ml^{-1}) during substrate incubations (laminarin, xylan, chondroitin sulphate) and unamended treatment control in the S. Gyre. Error bars indicate the total range of triplicates. b) Bar chart showing the bacterial community composition within biological triplicate substrate incubations and treatment control (not in triplicate) of the S. Gyre. All incubations were sampled at 0, 3, 6, 12 and 18 days.
