# Supplementary material for: Selfish, sharing and scavenging bacteria in the Atlantic Ocean: a biogeographical study of bacterial substrate utilisation
Source: ISME J. 2018 Dec 7;13(5):1119–32. doi: 10.1038/s41396-018-0326-3 (PMC6474216; doi:10.1038/s41396-018-0326-3)
Supplement: Supplementary file 13 — Supplementary Figure S10 [file 41396_2018_326_MOESM13_ESM.pdf]

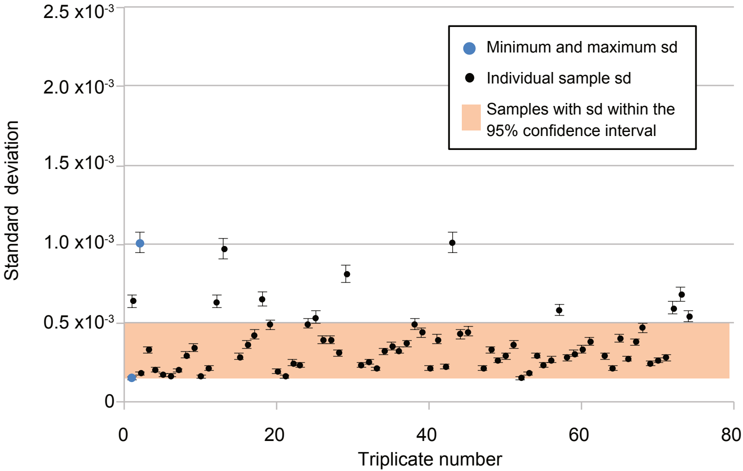

Supplementary Figure S10: Standard deviation (sd) and 95% confidence intervals (error bars) of the relative read abundance within biological triplicates. The minimum and maximum standard deviations of all triplicates are indicated by blue dots. Samples with a sd within the 95% confidence interval are highlighted in orange.
